# Poly[aqua(μ_3_-5-aza­niumylisophthalato)­(μ-oxalato)neodymium(III)]

**DOI:** 10.1107/S1600536812013554

**Published:** 2012-04-04

**Authors:** Xia Yin, Tian-Tian Xiao, Jun Fan, Sheng-Run Zheng, Wei-Guang Zhang

**Affiliations:** a School of Chemistry and Environment, South China Normal University, Guangzhou 510006, People’s Republic of China

## Abstract

The title compound, [Nd(C_8_H_6_NO_4_)(C_2_O_4_)(H_2_O)]_*n*_, is a layer-like coordination polymer. The Nd^III^ ion is coordinated by four carboxyl­ate O atoms from three bridging 5-aza­nium­yl­isophthalate (Haip) ligands, four carboxyl­ate O atoms from two oxalate (ox) anions and one ligated water mol­ecule in a tricapped trigonal–prismatic geometry. The Haip anion acts as a μ_3_-bridge, connecting three Nd^III^ ions through two carboxyl­ate groups; the ox anion adopts a bis-bidentate-bridging mode, linking two Nd^III^ ions. The layer framework is further extended to a three-dimensional supra­molecular structure through N—H⋯O and O—H⋯O hydrogen bonds.

## Related literature

For isotypic complexes, see: Liu *et al.* (2008 ▶); Yan *et al.* (2009 ▶).
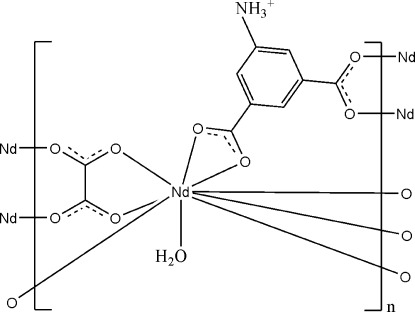


## Experimental

### 

#### Crystal data

[Nd(C_8_H_6_NO_4_)(C_2_O_4_)(H_2_O)]
*M* *_r_* = 430.41
Monoclinic, 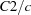

*a* = 20.047 (4) Å
*b* = 9.592 (2) Å
*c* = 13.670 (3) Å
β = 117.810 (2)°
*V* = 2325.0 (9) Å^3^
*Z* = 8
Mo *K*α radiation
μ = 4.52 mm^−1^
*T* = 298 K
0.28 × 0.22 × 0.15 mm

#### Data collection

Bruker APEXII CCD area-detector diffractometer
Absorption correction: multi-scan (*SADABS*; Bruker, 2002 ▶) *T*_min_ = 0.364, *T*_max_ = 0.551
5839 measured reflections
2104 independent reflections
1792 reflections with *I* > 2σ(*I*)
*R*_int_ = 0.033

#### Refinement

*R*[*F* ^2^ > 2σ(*F* ^2^)] = 0.024
*wR*(*F* ^2^) = 0.056
*S* = 1.03
2104 reflections
191 parameters
H-atom parameters constrained
Δρ_max_ = 0.71 e Å^−3^
Δρ_min_ = −0.53 e Å^−3^

### 

Data collection: *APEX2* (Bruker, 2007 ▶); cell refinement: *SAINT* (Bruker, 2007 ▶); data reduction: *SAINT*; program(s) used to solve structure: *SHELXS97* (Sheldrick, 2008 ▶); program(s) used to refine structure: *SHELXL97* (Sheldrick, 2008 ▶); molecular graphics: *SHELXTL* (Sheldrick, 2008 ▶); software used to prepare material for publication: *SHELXTL*.

## Supplementary Material

Crystal structure: contains datablock(s) I, global. DOI: 10.1107/S1600536812013554/ng5259sup1.cif

Structure factors: contains datablock(s) I. DOI: 10.1107/S1600536812013554/ng5259Isup2.hkl

Additional supplementary materials:  crystallographic information; 3D view; checkCIF report

## Figures and Tables

**Table 1:** Hydrogen-bond geometry (Å, °)

*D*—H⋯*A*	*D*—H	H⋯*A*	*D*⋯*A*	*D*—H⋯*A*
N1—H1*B*⋯O5^i^	0.89	1.91	2.795 (5)	172
N1—H1*C*⋯O8^ii^	0.89	2.05	2.872 (5)	154
O1*W*—H1*W*⋯O3^iii^	0.82	2.06	2.812 (4)	153
O1*W*—H2*W*⋯O1^iv^	0.82	1.97	2.750 (4)	159

Symmetry codes: (i) 
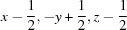
; (ii) 
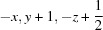
; (iii) 
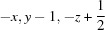
; (iv) 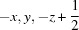.

**Fig. 1.**
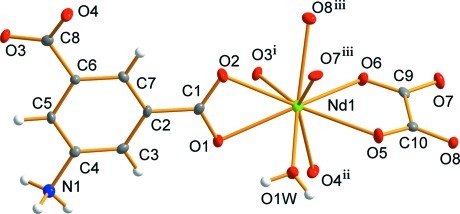
A fragment of the polymeric structure, with displacement ellipsoids drawn at the 30% probability level. [Symmetry codes: (i) -x, 1 - y, -z; (ii) x, -1 + y, z; (iii) 1/2 - x, 1/2 + y, 1/2 - z].

**Fig. 2.**
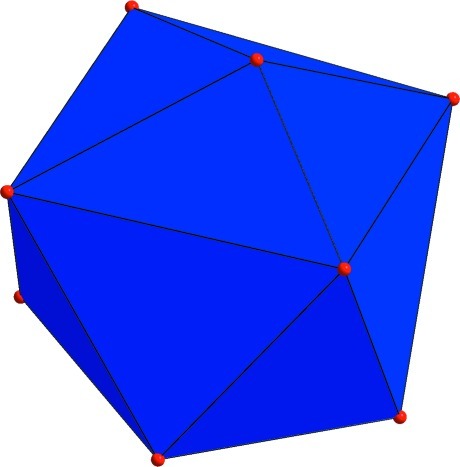
Geometry of the nine-coordinated NdIII ion in the title compound.

**Fig. 3.**
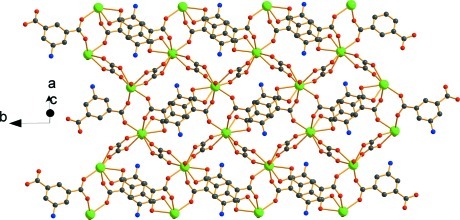
A packing diagram of the title compound, showing a layer-like structure in the ab plane.

**Fig. 4.**
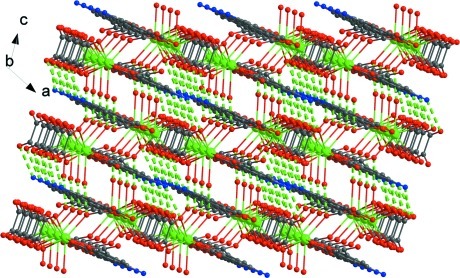
. A packing diagram, showing a three-dimensional supramolecular network driven by hydrogen bonds (dashed lines).

## supplementary crystallographic information

### Comment

The 5-aminoisophthalate (aip) anion adopts various coordination modes in lanthanide complexes. In this work, we present the synthesis and structure of a new neodymium coordination polymer with 5-aminoisophthalate and oxalate, [Nd(Haip)(ox)(H_2_O)], which is isostructural with those reported previously (Liu *et al.*, 2008; Yan *et al.*, 2009).

In the title compound, the asymmetric unit comprises one Nd^III^ ion, one Haip ligand, one oxalate anion and one ligated water molecule (Fig. 1). The neodymium ion is nine-coordinated by four carboxylate O atoms [O1, O2, O3^i^, and O4^ii^, symmetry codes: (i) -*x*, 1 - *y*, -*z*; (ii) *x*, -1 + *y*, *z*] from three Haip ligands, four carboxylate O atoms [O5, O6, O7^iii^, and O8^iii^, symmetry codes: (iii) 1/2 - *x*, 1/2 + *y*, 1/2 - *z*] from two oxalate ions and one coordinated water molecule. The geometry is a tricapped trigonal prism configuration (Fig. 2). The Nd—O bond distances are in the range of 2.417 (3)–2.603 (3) Å.

The Haip anion acts as *µ*_3_-bridge to connect three Nd^III^ ions through two carboxylate groups and the amino group exists as an –NH_3_^+^ unit. The oxalate anion adopts a bis-bidenatate-bridging mode to link two Nd^III^ ions with a Nd···Nd separation of 6.3821 (10) Å. The coordination of the metal ions and organic ligands (Haip and ox) results in the formation of a layer-like framework in the *ab* plane (Fig. 3).

In addition, there are O–H···O [O···O distances, 2.750 (4) and 2.812 (4) Å] and N–H···O hydrogen bonds (Table 1). The layers are further linked *via* these hydrogen bonds to form a three-dimensional supramolecular architecture (Fig. 4).

### Experimental

A mixture of 5-aminoisophthalic acid (0.50 mmol, 90.6 mg), Nd(NO_3_)_3_.6H_2_O (0.30 mmol, 131.5 mg,) oxalic acid (0.50 mmol, 45.0 mg) and 10 ml H_2_O was sealed in a 15 ml Teflon-lined stainless steel reactor and heated at 423 K under autogenous pressure for 72 h. After the sample had been slowly cooled to room temperature at a rate of 5 K/h, block-shaped pale-purple crystals were isolated (yield 52%). IR (KBr pellet, ν cm^-1^): 3423 (*m*), 1631 (*s*), 1570 (*s*), 1466 (*m*), 1394 (*s*), 1326 (*m*), 1116 (*m*), 914 (*w*), 769 (*s*), 596 (*m*).

### Refinement

The H atoms of water molecule were located in a difference Fourier maps and the others were placed in calculated positions and refined as riding atoms with isotropic thermal factors [C–H = 0.93 (aromatic C–H) Å; N–H = 0.89 Å; O–H = 0.83 Å; *U*_iso_(H) = 1.2 *U*_eq_ (C), *U*_iso_(H) = 1.5*U*_eq_(N), and *U*_iso_(H) = 1.5*U*_eq_(O)].

### Crystal data

**Table:**

[Nd(C_8_H_6_NO_4_)(C_2_O_4_)(H_2_O)]	*F*(000) = 1656
*M**_r_* = 430.41	*D*_x_ = 2.459 Mg m^−^^3^
Monoclinic, *C*2/*c*	Mo *K*α radiation, λ = 0.71073 Å
Hall symbol: -c 2yc	Cell parameters from 2699 reflections
*a* = 20.047 (4) Å	θ = 2.3–28.1°
*b* = 9.592 (2) Å	µ = 4.52 mm^−^^1^
*c* = 13.670 (3) Å	*T* = 298 K
β = 117.810 (2)°	Block, pale-purple
*V* = 2325.0 (9) Å^3^	0.28 × 0.22 × 0.15 mm
*Z* = 8	

### Data collection

**Table:**

Bruker APEXII CCD area-detector diffractometer	2104 independent reflections
Radiation source: fine-focus sealed tube	1792 reflections with *I* > 2σ(*I*)
Graphite monochromator	*R*_int_ = 0.033
φ and ω scan	θ_max_ = 25.3°, θ_min_ = 2.3°
Absorption correction: multi-scan (*SADABS*; Bruker, 2002)	*h* = −24→23
*T*_min_ = 0.364, *T*_max_ = 0.551	*k* = −6→11
5839 measured reflections	*l* = −16→15

### Refinement

**Table:**

Refinement on *F*^2^	Primary atom site location: structure-invariant direct methods
Least-squares matrix: full	Secondary atom site location: difference Fourier map
*R*[*F*^2^ > 2σ(*F*^2^)] = 0.024	Hydrogen site location: inferred from neighbouring sites
*wR*(*F*^2^) = 0.056	H-atom parameters constrained
*S* = 1.03	*w* = 1/[σ^2^(*F*_o_^2^) + (0.0254*P*)^2^] where *P* = (*F*_o_^2^ + 2*F*_c_^2^)/3
2104 reflections	(Δ/σ)_max_ < 0.001
191 parameters	Δρ_max_ = 0.71 e Å^−^^3^
0 restraints	Δρ_min_ = −0.53 e Å^−^^3^

### Special details

**Table:**

Geometry. All e.s.d.'s (except the e.s.d. in the dihedral angle between two l.s. planes) are estimated using the full covariance matrix. The cell e.s.d.'s are taken into account individually in the estimation of e.s.d.'s in distances, angles and torsion angles; correlations between e.s.d.'s in cell parameters are only used when they are defined by crystal symmetry. An approximate (isotropic) treatment of cell e.s.d.'s is used for estimating e.s.d.'s involving l.s. planes.
Refinement. Refinement of *F*^2^ against ALL reflections. The weighted *R*-factor *wR* and goodness of fit *S* are based on *F*^2^, conventional *R*-factors *R* are based on *F*, with *F* set to zero for negative *F*^2^. The threshold expression of *F*^2^ > σ(*F*^2^) is used only for calculating *R*-factors(gt) *etc*. and is not relevant to the choice of reflections for refinement. *R*-factors based on *F*^2^ are statistically about twice as large as those based on *F*, and *R*- factors based on ALL data will be even larger.

### Fractional atomic coordinates and isotropic or equivalent isotropic displacement parameters (Å^2^)

**Table:**

	*x*	*y*	*z*	*U*_iso_*/*U*_eq_	
C1	0.0410 (3)	0.3120 (5)	0.1508 (3)	0.0193 (10)	
C2	−0.0121 (3)	0.4328 (4)	0.1225 (4)	0.0191 (10)	
C3	−0.0873 (3)	0.4068 (5)	0.0894 (3)	0.0202 (10)	
H3	−0.1043	0.3155	0.0846	0.024*	
C4	−0.1372 (2)	0.5164 (4)	0.0632 (3)	0.0172 (10)	
C5	−0.1136 (2)	0.6520 (4)	0.0653 (3)	0.0184 (10)	
H5	−0.1480	0.7249	0.0465	0.022*	
C6	−0.0386 (2)	0.6794 (4)	0.0956 (3)	0.0154 (9)	
C7	0.0131 (2)	0.5702 (4)	0.1271 (3)	0.0181 (10)	
H7	0.0639	0.5882	0.1511	0.022*	
C8	−0.0168 (3)	0.8255 (5)	0.0841 (4)	0.0200 (10)	
C9	0.2271 (2)	−0.2176 (5)	0.1774 (4)	0.0180 (10)	
C10	0.2346 (2)	−0.2223 (5)	0.2950 (4)	0.0191 (10)	
N1	−0.2164 (2)	0.4895 (4)	0.0294 (3)	0.0238 (9)	
H1A	−0.2248	0.3981	0.0228	0.036*	
H1B	−0.2448	0.5306	−0.0353	0.036*	
H1C	−0.2282	0.5235	0.0800	0.036*	
Nd1	0.131834 (12)	0.06274 (2)	0.184227 (18)	0.01456 (9)	
O1	0.01407 (17)	0.1908 (3)	0.1433 (3)	0.0254 (7)	
O2	0.10891 (17)	0.3304 (3)	0.1781 (2)	0.0278 (8)	
O3	−0.06874 (17)	0.9033 (3)	0.0145 (2)	0.0203 (7)	
O4	0.05001 (17)	0.8623 (3)	0.1427 (3)	0.0258 (8)	
O5	0.20791 (17)	−0.1221 (3)	0.3242 (2)	0.0232 (7)	
O6	0.19047 (18)	−0.1178 (3)	0.1175 (2)	0.0240 (7)	
O7	0.25959 (17)	−0.3114 (3)	0.1530 (2)	0.0264 (8)	
O8	0.26728 (17)	−0.3267 (3)	0.3532 (2)	0.0235 (7)	
O1W	0.10490 (18)	0.0638 (3)	0.3446 (3)	0.0276 (8)	
H1W	0.1092	0.0089	0.3930	0.041*	
H2W	0.0717	0.1186	0.3396	0.041*	

### Atomic displacement parameters (Å^2^)

**Table:**

	*U*^11^	*U*^22^	*U*^33^	*U*^12^	*U*^13^	*U*^23^
C1	0.026 (3)	0.018 (3)	0.015 (2)	0.004 (2)	0.011 (2)	0.0035 (19)
C2	0.023 (2)	0.017 (3)	0.019 (2)	0.008 (2)	0.010 (2)	0.0066 (19)
C3	0.025 (3)	0.018 (3)	0.018 (2)	−0.0024 (19)	0.011 (2)	0.0026 (19)
C4	0.019 (2)	0.015 (2)	0.016 (2)	−0.0019 (19)	0.0066 (19)	−0.0008 (19)
C5	0.018 (2)	0.014 (2)	0.022 (2)	0.0044 (18)	0.008 (2)	0.0010 (19)
C6	0.015 (2)	0.015 (2)	0.017 (2)	−0.0006 (18)	0.0076 (19)	0.0005 (19)
C7	0.014 (2)	0.021 (3)	0.018 (2)	0.0002 (19)	0.0065 (19)	−0.0005 (19)
C8	0.025 (3)	0.017 (3)	0.021 (2)	−0.002 (2)	0.014 (2)	−0.002 (2)
C9	0.010 (2)	0.018 (3)	0.023 (2)	−0.0036 (18)	0.007 (2)	−0.006 (2)
C10	0.016 (2)	0.019 (3)	0.023 (2)	−0.0017 (19)	0.010 (2)	0.002 (2)
N1	0.023 (2)	0.018 (2)	0.028 (2)	−0.0009 (17)	0.0108 (18)	−0.0036 (17)
Nd1	0.01452 (14)	0.01069 (14)	0.01771 (14)	0.00055 (10)	0.00688 (10)	0.00072 (10)
O1	0.0239 (18)	0.0142 (18)	0.040 (2)	0.0040 (14)	0.0170 (16)	0.0043 (15)
O2	0.0174 (18)	0.0239 (19)	0.039 (2)	0.0046 (14)	0.0104 (15)	0.0028 (15)
O3	0.0251 (18)	0.0131 (17)	0.0175 (16)	0.0024 (13)	0.0055 (14)	0.0022 (13)
O4	0.0208 (18)	0.0158 (18)	0.0324 (18)	−0.0050 (14)	0.0054 (15)	0.0014 (14)
O5	0.0280 (19)	0.0191 (17)	0.0234 (17)	0.0092 (14)	0.0128 (15)	−0.0022 (14)
O6	0.0337 (19)	0.0192 (18)	0.0240 (18)	0.0091 (15)	0.0175 (16)	0.0057 (14)
O7	0.0238 (18)	0.027 (2)	0.0259 (17)	0.0093 (15)	0.0096 (15)	−0.0052 (15)
O8	0.0238 (18)	0.0200 (19)	0.0296 (18)	0.0066 (14)	0.0149 (15)	0.0089 (14)
O1W	0.0317 (19)	0.028 (2)	0.0304 (19)	0.0082 (15)	0.0204 (16)	0.0084 (14)

### Geometric parameters (Å, º)

**Table:**

C1—O2	1.245 (5)	C9—C10	1.544 (6)
C1—O1	1.265 (5)	C10—O5	1.253 (5)
C1—C2	1.498 (6)	C10—O8	1.256 (5)
C2—C3	1.379 (6)	N1—H1A	0.8900
C2—C7	1.402 (6)	N1—H1B	0.8900
C3—C4	1.379 (6)	N1—H1C	0.8900
C3—H3	0.9300	Nd1—O4^i^	2.417 (3)
C4—C5	1.380 (6)	Nd1—O3^ii^	2.425 (3)
C4—N1	1.454 (5)	Nd1—O1	2.482 (3)
C5—C6	1.387 (6)	Nd1—O1W	2.491 (3)
C5—H5	0.9300	Nd1—O6	2.492 (3)
C6—C7	1.392 (6)	Nd1—O5	2.532 (3)
C6—C8	1.497 (6)	Nd1—O8^iii^	2.538 (3)
C7—H7	0.9300	Nd1—O7^iii^	2.575 (3)
C8—O4	1.248 (5)	Nd1—O2	2.603 (3)
C8—O3	1.274 (5)	O1W—H1W	0.8182
C9—O7	1.244 (5)	O1W—H2W	0.8249
C9—O6	1.252 (5)		
			
O2—C1—O1	121.3 (4)	O4^i^—Nd1—O6	75.26 (11)
O2—C1—C2	120.9 (4)	O3^ii^—Nd1—O6	76.82 (10)
O1—C1—C2	117.7 (4)	O1—Nd1—O6	145.34 (10)
C3—C2—C7	120.1 (4)	O1W—Nd1—O6	131.15 (10)
C3—C2—C1	118.7 (4)	O4^i^—Nd1—O5	73.98 (10)
C7—C2—C1	121.2 (4)	O3^ii^—Nd1—O5	139.16 (10)
C4—C3—C2	119.8 (4)	O1—Nd1—O5	133.70 (10)
C4—C3—H3	120.1	O1W—Nd1—O5	68.83 (10)
C2—C3—H3	120.1	O6—Nd1—O5	64.51 (9)
C3—C4—C5	120.9 (4)	O4^i^—Nd1—O8^iii^	143.36 (10)
C3—C4—N1	120.0 (4)	O3^ii^—Nd1—O8^iii^	76.48 (10)
C5—C4—N1	119.1 (4)	O1—Nd1—O8^iii^	120.75 (10)
C4—C5—C6	119.9 (4)	O1W—Nd1—O8^iii^	134.10 (10)
C4—C5—H5	120.0	O6—Nd1—O8^iii^	70.15 (10)
C6—C5—H5	120.0	O5—Nd1—O8^iii^	100.92 (10)
C5—C6—C7	119.7 (4)	O4^i^—Nd1—O7^iii^	141.60 (10)
C5—C6—C8	118.4 (4)	O3^ii^—Nd1—O7^iii^	133.95 (10)
C7—C6—C8	121.7 (4)	O1—Nd1—O7^iii^	107.11 (10)
C6—C7—C2	119.5 (4)	O1W—Nd1—O7^iii^	71.34 (10)
C6—C7—H7	120.2	O6—Nd1—O7^iii^	106.82 (10)
C2—C7—H7	120.2	O5—Nd1—O7^iii^	72.95 (10)
O4—C8—O3	124.9 (4)	O8^iii^—Nd1—O7^iii^	63.03 (10)
O4—C8—C6	118.4 (4)	O4^i^—Nd1—O2	133.39 (10)
O3—C8—C6	116.7 (4)	O3^ii^—Nd1—O2	80.76 (9)
O7—C9—O6	126.7 (4)	O1—Nd1—O2	50.92 (10)
O7—C9—C10	116.8 (4)	O1W—Nd1—O2	85.21 (10)
O6—C9—C10	116.5 (4)	O6—Nd1—O2	141.25 (10)
O5—C10—O8	125.7 (4)	O5—Nd1—O2	138.60 (9)
O5—C10—C9	117.5 (4)	O8^iii^—Nd1—O2	74.13 (10)
O8—C10—C9	116.8 (4)	O7^iii^—Nd1—O2	68.31 (10)
C4—N1—H1A	109.5	C1—O1—Nd1	96.4 (3)
C4—N1—H1B	109.5	C1—O2—Nd1	91.2 (3)
H1A—N1—H1B	109.5	C8—O3—Nd1^ii^	137.1 (3)
C4—N1—H1C	109.5	C8—O4—Nd1^iv^	141.5 (3)
H1A—N1—H1C	109.5	C10—O5—Nd1	119.5 (3)
H1B—N1—H1C	109.5	C9—O6—Nd1	121.7 (3)
O4^i^—Nd1—O3^ii^	84.35 (10)	C9—O7—Nd1^v^	116.4 (3)
O4^i^—Nd1—O1	82.57 (10)	C10—O8—Nd1^v^	115.8 (3)
O3^ii^—Nd1—O1	74.75 (10)	Nd1—O1W—H1W	136.4
O4^i^—Nd1—O1W	78.94 (10)	Nd1—O1W—H2W	115.3
O3^ii^—Nd1—O1W	140.51 (11)	H1W—O1W—H2W	104.8
O1—Nd1—O1W	67.81 (10)		
			
O2—C1—C2—C3	178.0 (4)	O4^i^—Nd1—O2—C1	2.9 (3)
O1—C1—C2—C3	−0.4 (6)	O3^ii^—Nd1—O2—C1	76.0 (3)
O2—C1—C2—C7	−0.7 (7)	O1—Nd1—O2—C1	−1.9 (2)
O1—C1—C2—C7	−179.0 (4)	O1W—Nd1—O2—C1	−67.0 (3)
C7—C2—C3—C4	−1.5 (7)	O6—Nd1—O2—C1	131.1 (3)
C1—C2—C3—C4	179.8 (4)	O5—Nd1—O2—C1	−117.0 (3)
C2—C3—C4—C5	2.7 (7)	O8^iii^—Nd1—O2—C1	154.4 (3)
C2—C3—C4—N1	−179.3 (4)	O7^iii^—Nd1—O2—C1	−138.8 (3)
C3—C4—C5—C6	−1.0 (6)	O4—C8—O3—Nd1^ii^	100.8 (5)
N1—C4—C5—C6	−178.9 (4)	C6—C8—O3—Nd1^ii^	−79.5 (5)
C4—C5—C6—C7	−2.1 (6)	O3—C8—O4—Nd1^iv^	2.2 (8)
C4—C5—C6—C8	172.9 (4)	C6—C8—O4—Nd1^iv^	−177.5 (3)
C5—C6—C7—C2	3.3 (6)	O8—C10—O5—Nd1	−173.2 (3)
C8—C6—C7—C2	−171.5 (4)	C9—C10—O5—Nd1	7.0 (5)
C3—C2—C7—C6	−1.5 (7)	O4^i^—Nd1—O5—C10	75.8 (3)
C1—C2—C7—C6	177.2 (4)	O3^ii^—Nd1—O5—C10	15.1 (4)
C5—C6—C8—O4	156.3 (4)	O1—Nd1—O5—C10	138.4 (3)
C7—C6—C8—O4	−28.9 (6)	O1W—Nd1—O5—C10	159.9 (3)
C5—C6—C8—O3	−23.5 (6)	O6—Nd1—O5—C10	−5.2 (3)
C7—C6—C8—O3	151.4 (4)	O8^iii^—Nd1—O5—C10	−66.8 (3)
O7—C9—C10—O5	173.9 (4)	O7^iii^—Nd1—O5—C10	−124.0 (3)
O6—C9—C10—O5	−4.4 (6)	O2—Nd1—O5—C10	−145.2 (3)
O7—C9—C10—O8	−6.0 (6)	O7—C9—O6—Nd1	−178.6 (3)
O6—C9—C10—O8	175.8 (4)	C10—C9—O6—Nd1	−0.6 (5)
O2—C1—O1—Nd1	−3.5 (4)	O4^i^—Nd1—O6—C9	−76.3 (3)
C2—C1—O1—Nd1	174.9 (3)	O3^ii^—Nd1—O6—C9	−163.8 (3)
O4^i^—Nd1—O1—C1	−174.7 (3)	O1—Nd1—O6—C9	−128.3 (3)
O3^ii^—Nd1—O1—C1	−88.6 (3)	O1W—Nd1—O6—C9	−15.8 (4)
O1W—Nd1—O1—C1	104.3 (3)	O5—Nd1—O6—C9	2.7 (3)
O6—Nd1—O1—C1	−124.5 (3)	O8^iii^—Nd1—O6—C9	116.0 (3)
O5—Nd1—O1—C1	125.9 (2)	O7^iii^—Nd1—O6—C9	63.8 (3)
O8^iii^—Nd1—O1—C1	−24.9 (3)	O2—Nd1—O6—C9	139.9 (3)
O7^iii^—Nd1—O1—C1	43.4 (3)	O6—C9—O7—Nd1^v^	156.9 (4)
O2—Nd1—O1—C1	1.8 (2)	C10—C9—O7—Nd1^v^	−21.2 (5)
O1—C1—O2—Nd1	3.3 (4)	O5—C10—O8—Nd1^v^	−149.6 (4)
C2—C1—O2—Nd1	−175.0 (4)	C9—C10—O8—Nd1^v^	30.3 (5)

Symmetry codes: (i) *x*, *y*−1, *z*; (ii) −*x*, −*y*+1, −*z*; (iii) −*x*+1/2, *y*+1/2, −*z*+1/2; (iv) *x*, *y*+1, *z*; (v) −*x*+1/2, *y*−1/2, −*z*+1/2.

### Hydrogen-bond geometry (Å, º)

**Table:**

*D*—H···*A*	*D*—H	H···*A*	*D*···*A*	*D*—H···*A*
N1—H1*B*···O5^vi^	0.89	1.91	2.795 (5)	172
N1—H1*C*···O8^vii^	0.89	2.05	2.872 (5)	154
O1*W*—H1*W*···O3^viii^	0.82	2.06	2.812 (4)	153
O1*W*—H2*W*···O1^ix^	0.82	1.97	2.750 (4)	159

Symmetry codes: (vi) *x*−1/2, −*y*+1/2, *z*−1/2; (vii) −*x*, *y*+1, −*z*+1/2; (viii) −*x*, *y*−1, −*z*+1/2; (ix) −*x*, *y*, −*z*+1/2.
